# Supplementary material for: Chromothripsis during telomere crisis is independent of NHEJ, and consistent with a replicative origin
Source: Genome Res. 2019 May;29(5):737–49. doi: 10.1101/gr.240705.118 (PMC6499312; doi:10.1101/gr.240705.118)
Supplement: Supplemental Material [file supp_gr.240705.118_Supplemental_file_1.zip › contigs/annotated_contigs/DB79/contig.3.DB79_length_457_mean_cov_6.31509846827.docx]

**DB79_length_457_mean_cov_6.31509846827**

```
GGTTGCAAACCTACCAAGAATGCATAATAGTCTCACTTTTCCCCAATAAAGAGATGCGTGTGACTAGTTTTGGACTTTTAACCTTAATG
 >chr1:35644676-35644949 - E=8e-153
GGGGTTGCATGTCTCCTATTGTTAATCATTGTCAGCTGCAGTGACATGATCCACAGTCCTGCATTTACTGCCTTTCACTTAATGATTTT

GGACAGGTTTTAGAGAGCCAGGATGTTGGTCTGGGCCTTTATTTGGTTTTGGCTTTAGCTGATAATGTTTTAGTGATGTCAGCTAGTGC

AG|TTCT|TGTATTTTAGTAGATACGGGGTTTCACCCTATTGCCCAGGCTGGTCTCGAACTCCTGAGCTCAGGCAATCCACCCACCTTG
   >chr1:35710338-35710477 - E=4e-72
GCCTCCCAAAGTGCTGGGATTACAGGTGTGAGCCACCGCACCTGGCCT|AAATTT|ATATGGATTGTGAGTCTACAAAATACCACTGGA
                                                 >chr1:239556256-239556311 - E=2e-21
TTAGGTTTCAATGGAA
```
